# Long term T cell response and safety of a tetravalent dengue vaccine in healthy children

**DOI:** 10.1038/s41541-024-00967-0

**Published:** 2024-10-17

**Authors:** Sanja Mandaric, Heather Friberg, Xavier Saez-Llorens, Charissa Borja-Tabora, Shibadas Biswal, Ian Escudero, Alice Faccin, Raphael Gottardo, Manja Brose, Nicholas Roubinis, Darlene Fladager, Rodrigo DeAntonio, Julie Anne L. Dimero, Nathali Montenegro, Nicolas Folschweiller, Jeffrey R. Currier, Mayuri Sharma, Vianney Tricou

**Affiliations:** 1 Takeda Pharmaceuticals International AG, Zurich, Switzerland; 2 Walter Reed Army Institute of Research, Silver Spring, MD USA; 3 Hospital del Niño Dr. José Renán Esquivel, Panama City, Panama; 4 Centro de Vacunación Internacional Cevaxin, Panama City, Panama; 5 Sistema Nacional de Investigación SENACYT, Panama City, Panama; 6 Research Institute for Tropical Medicine, Muntinlupa, Philippines; 7 Takeda Vaccines, Inc., Cambridge, MA USA; 8 Lausanne University Hospital and University of Lausanne, Lausanne, Switzerland

**Keywords:** Viral infection, Drug development, Phase II trials

## Abstract

As robust cellular responses are important for protection against dengue, this phase 2 study evaluated the kinetics and phenotype of T cell responses induced by TAK-003, a live-attenuated tetravalent dengue vaccine, in 4–16-year-old living in dengue-endemic countries (NCT02948829). Two hundred participants received TAK-003 on Days 1 and 90. Interferon-gamma (IFN-γ) enzyme-linked immunospot assay [ELISPOT] and intracellular cytokine staining were used to analyze T cell response and functionality, using peptide pools representing non-structural (NS) proteins NS3 and NS5 matching DENV-1, -2, -3, and -4 and DENV-2 NS1. One month after the second TAK-003 dose (Day 120), IFN-γ ELISPOT T cell response rates against any peptide pool were 97.1% (95% CI: 93.4% to 99.1%), and similar for baseline dengue seropositive (96.0%) and seronegative (98.6%) participants. IFN-γ ELISPOT T cell response rates at Day 120 were 79.8%, 90.2%, 77.3%, and 74.0%, against DENV-1, -2, -3, and -4, respectively, and remained elevated through 3 years post-vaccination. Multifunctional CD4 and CD8 T cell responses against DENV-2 NS peptides were observed, independent of baseline serostatus: CD8 T cells typically secreted IFN-γ and TNF-α whereas CD4 T cells secreted ≥ 2 of IFN-γ, IL-2 and TNF-α cytokines. NAb titers and seropositivity rates remained substantially elevated through 3 years post-vaccination. Overall, TAK-003 was well tolerated and elicited durable T cell responses against all four DENV serotypes irrespective of baseline serostatus in children and adolescents aged 4–16 years living in dengue-endemic countries. TAK-003-elicited CD4 and CD8 T cells were multifunctional and persisted up to 3 years post-vaccination.

## Introduction

Dengue (DENV), a mosquito-borne *Flavivirus* that is primarily found in tropical and subtropical regions, is responsible for approximately 390 million infections annually, with a rapid increase in cases reported in recent decades^1,2^. Currently, more than 100 countries are endemic for dengue, a number which is likely to increase in coming years as mosquito vectors such as *Aedes aegypti* spread into more temperate and higher altitude regions related to social and environmental factors including urbanization, climate change, and globalization^2,3^. While the majority of individuals who are infected with dengue experience mild or no symptoms, approximately 5% of patients with symptomatic disease develop severe dengue, with approximately 22,000 deaths occurring annually, mainly in children^4,5^. Infection by one of the four DENV serotypes is thought to provide lifelong homotypic immunity against the same DENV serotype, however, individuals remain susceptible to infection by the other DENV serotypes. Heterotypic secondary infection is associated with higher rates of severe outcomes and hospitalizations, likely owing at least in part to antibody-dependent enhancement in addition to the infecting serotype^6,7^, although recent data have shown that primary infections still account for more than half of clinical and severe dengue cases^8^.

Considering the importance of DENV-specific T cells in protective immunity from dengue^9–14^, an ideal vaccine would elicit both cellular and humoral immunity against all four dengue serotypes simultaneously, together with providing long-term disease protection and no evidence of disease enhancement. Understanding the nature of vaccine-elicited T cell responses is critical for the evaluation of potential vaccines, and as such, evaluation of cellular responses is recommended by the World Health Organization for assessment of candidate dengue vaccines for use in endemic areas, as it can provide information on both immunological memory and the durability of vaccine responses^15,16^. In natural dengue infections, immunodominant targets for CD8 T cells are primarily dengue non-structural (NS) proteins NS3 and NS5 whereas CD4 T cells target mainly capsid, envelope, NS3, NS2A/B, and NS5 proteins^9,13^. Vigorous polyfunctional CD8 T cell responses for human leukocyte antigen alleles were associated with a reduced risk of severe dengue, indicating an important role of DENV-specific T cells in protective immunity^14^. While murine models have shown that antibody titers and CD8 T cell responses are seemingly not influenced by CD4 T cell numbers^11^, it is likely that CD4 T cells also contribute to viral clearance, as the control of dengue infection in asymptomatic individuals has been shown to correlate with numbers of activated CD4 T cells^12^. Therefore, both CD4 and CD8 T cell responses following vaccination, together with the induction of neutralizing antibodies, all contribute to a vaccine’s ability to provide protection.

TAK-003 is a live-attenuated tetravalent dengue vaccine based on a DENV-2 backbone. The virus for serotype 2 is a molecularly-characterized, laboratory-derived, attenuated DENV-2 virus. Recombinant chimeric viruses for serotypes 1, 3, and 4 were generated by substituting the structural membrane and envelope genes with those from DENV-1, DENV-3, and DENV-4 strains^17^. TAK-003 has demonstrated safety and efficacy against symptomatic and hospitalized dengue in a phase 3 study in children and adolescents living in dengue-endemic regions of Asia and Latin America^18–22^, and is currently approved in Argentina, Brazil, Colombia, Indonesia, Israel, Malaysia, Switzerland, Thailand, Vietnam, the European Union, the European Economic Area, and the United Kingdom for use without the need for prior serostatus testing. The backbone of TAK-003 elicits immune responses against DENV-specific NS proteins, with NS1, NS3, and NS5 previously identified as the immunodominant targets of the TAK-003-mediated T cell response^23–25^. Data from phase 1 studies of early formulations of TAK-003 showed potent and durable T cell responses against both structural and NS1, NS3, and NS5 components of all four DENV serotypes. These responses are predominantly mediated by interferon-gamma (IFN-γ)-producing CD8 T cells, with some additional DENV-specific CD4 T cell responses^23,24,26^, with the magnitude of IFN-γ ELISPOT responses to DENV-1, -3, and -4 NS proteins directly proportional to the magnitude of the corresponding DENV-2 NS protein response^23^. Exploratory analysis of data from a phase 2 study of three different dose schedules (months 0 and 3; months 0 and 12; and month 0 only) of TAK-003 in adolescents enrolled in Panama also demonstrated potent and durable multifactorial T cell responses, which were cross-reactive against DENV-1, DENV-3, and DENV-4 independent of baseline serostatus^25^.

Based on the findings of these exploratory analyses, we have conducted a long-term phase 2 study specifically focused on the analysis of T cell responses and safety following TAK-003 vaccination of children and adolescents living in Panama and the Philippines. The study population was chosen to complement the immunogenicity, safety, and efficacy data from the long-term pivotal phase 3 study of two doses of TAK-003 administered 3 months apart, conducted in healthy participants aged 4–16 years living in dengue-endemic areas of Asia and Latin America (NCT02747927). The current study comprised a similar study population in terms of age (i.e., 4–16 years), region (i.e., Asia and Latin America), and enrollment timeframe (2016–2017), and evaluates T cell responses up to 3 years after the second vaccine dose in terms of magnitude, kinetics, and durability of response. Further analyses assessed T cell response serotype coverage, multifunctional T cell capacity, neutralizing antibody (NAb) and T cell responses in TAK-003 recipients who developed breakthrough dengue, and any age/regional effects on T cell responses.

## Results

**Fig. 1:**
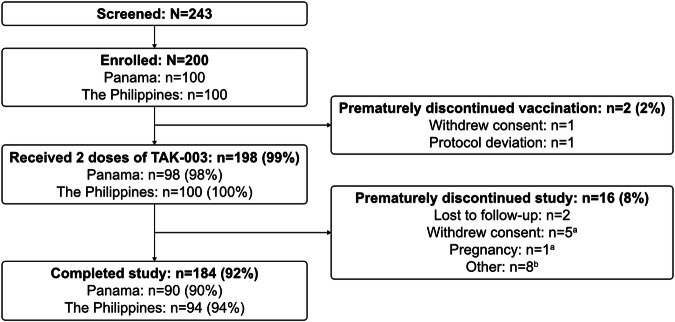
Participant flow. Numbers of participants who completed the study in each country, and reasons for discontinuation. ^a^Participants enrolled in Panama; ^b^six participants enrolled in the Philippines and two participants enrolled in Panama.

In total, 200 participants were enrolled in the study (100 in each country), of whom 198 (99.0%) received two doses of TAK-003. The other two participants only received one dose of TAK-003 due to withdrawal of consent or poor adherence to trial procedures (Fig. 1). Overall, 195 participants were included in the per-protocol set (PPS) (Table 1), and 184 (92%) participants completed the study through to 3 years post-vaccination. The mean age of participants was 6.6 years at baseline, and 56% were seropositive. In total, 79 participants met the criteria for inclusion in the intracellular cytokine staining (ICS) subset.

**Table 1:** Baseline demographics in each of the two study countries (per protocol set)

Characteristic	Panama, (*N* = 97)	Philippines, (*N* = 98)	Total, (*N* = 195)
Age, mean (SD), *y*	7.3 (2.85)	6.0 (1.24)	6.6 (2.29)
Age category, *n* (%)			
4–5 years	34 (35.1)	35 (35.7)	69 (35.4)
6–11 years	53 (54.6)	63 (64.3)	116 (59.5)
12–16 years	10 (10.3)	0 (0.0)	10 (5.1)
Sex, *n* (%)			
Male	47 (48.5)	49 (50.0)	96 (49.2)
Female	50 (51.5)	49 (50.0)	99 (50.8)
Race, *n* (%)			
American Indian or Alaska native	73 (75.3)	0 (0.0)	73 (37.4)
Asian	0 (0.0)	98 (100.0)	98 (50.3)
Black or African American	8 (8.2)	0 (0.0)	8 (4.1)
White	9 (9.3)	0 (0.0)	9 (4.6)
Multiracial	7 (7.2)	0 (0.0)	7 (3.6)
Body mass index, mean (SD), kg/m^2^	16.6 (3.82)	15.8 (2.18)	16.2 (3.13)
Baseline seropositivity status, *n* (%)			
Seropositive	45 (46.4)	64 (65.3)	109 (55.9)
Seronegative	52 (53.6)	34 (34.7)	86 (44.1)

*SD* standard deviation.

### T cell responses

One month after the second dose (Day 120), IFN-γ ELISPOT T cell response rates to any peptide pool were 97.1% (95% confidence interval (CI): 93.4% to 99.1%) for the entire study population (Table 2). Rates were similar irrespective of baseline serostatus (seropositive: 96.0% and seronegative: 98.6%), country (Panama: 97.3% and Philippines: 96.9%), or age group (4–5 years: 98.3%; 6–11 years: 96.2%; and 12–16 years: 100%; Table 2).

**Table 2:** T cell response rates against DENV one month after the second dose of TAK-003 (Day 120) for the total study population and stratified by baseline serostatus, country, and age group at enrollment (per protocol set)

Population	*n*/*N*	Response rate, (95% CI)
All participants	168/173	97.1% (93.4–99.1)
Baseline serostatus		
Seropositive	96/100	96.0% (90.1–98.9)
Seronegative	72/73	98.6% (92.6–100)
Country		
Panama	73/75	97.3% (90.7–99.7)
Philippines	95/98	96.9% (91.3–99.4)
Age group		
4–5 years	59/60	98.3% (91.1–100)
6–11 years	100/104	96.2% (90.4–98.9)
12–16 years	9/9	100% (66.4–100)

A positive T cell response was defined as IFN-γ ELISPOT response >3 times higher than baseline and ≥5 spot-forming cells (SFC)/10^6^ peripheral blood mononuclear cells. Limit of detection (LOD): 5 SFC/10^6^ PBMC. Peptide pools evaluated: DENV-2 NS1; DENV-1, -2, -3, and -4 NS3, and DENV-1, -2, -3, and -4 NS5.

*n* number of positive responders, *N* total number of participants evaluated.

**Fig. 2:**
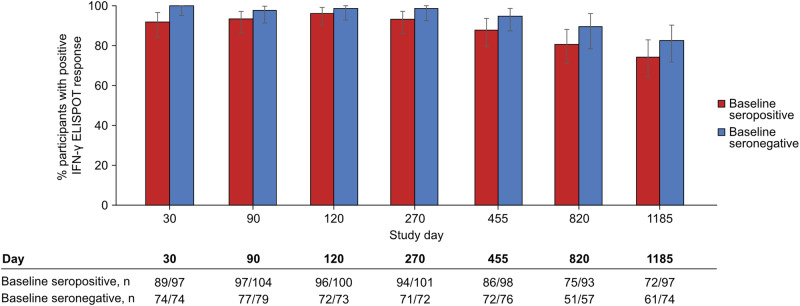
T cell response rates against DENV induced by TAK-003. Percentage (error bars: 95% CI) of participants with a positive T cell response to any DENV peptide pool from first vaccination to Day 1185, by baseline serostatus (per protocol set). The percentage of positive responders is represented as the overall percentage and corresponding 95% CI, with a number of responders/total number of participants with samples included in the table. Peptide pools evaluated: DENV-2 NS1; DENV-1, -2, -3, and -4 NS3, and DENV-1, -2, -3, and -4 NS5. A positive T cell response was defined as IFN-γ ELISPOT response >3 times higher than baseline and ≥5 spot forming cells [SFCs]/10^6^ peripheral blood mononuclear cells [PBMCs]). LOD: 5 SFC/10^6^ PBMC. The terms “seropositive” and “seronegative” refer to baseline dengue serostatus. Seropositivity was defined as a neutralizing titer MNT ≥ 10 for at least one DENV serotype. ELISPOT enzyme-linked immunospot, IFN-γ interferon-gamma.

IFN-γ ELISPOT T cell response rates persisted at high levels throughout the study in both seropositive and seronegative participants (Fig. 2). In baseline seropositive participants, the response rate to any peptide pool was 91.8% at Day 30, 93.3% at Day 90, 96.0% at Day 120, 93.1% at Day 270, 87.8% at Year 1, 80.6% at Year 2, and 74.2% at Year 3, while in baseline seronegative participants response rates were 100.0% at Day 30, 97.5% at Day 90, 98.6% at Day 120, 98.6% at Day 270, 94.7% at Year 1, 89.5% at Year 2, and 82.4% at Year 3. In the subgroup of participants > 10 years of age with data for Day 14, response rates to any peptide pool were 85.7% (*n* = 6/7) and 33.3% (*n* = 1/3) for seropositive and seronegative participants, respectively. Similar trends in response rates over the three-year study period were seen across regions (Supplementary Fig. 1a) and age groups (Supplementary Fig. 1b), although it should be noted that there were very few participants in the 12–16-year-old age group.

**Fig. 3:**
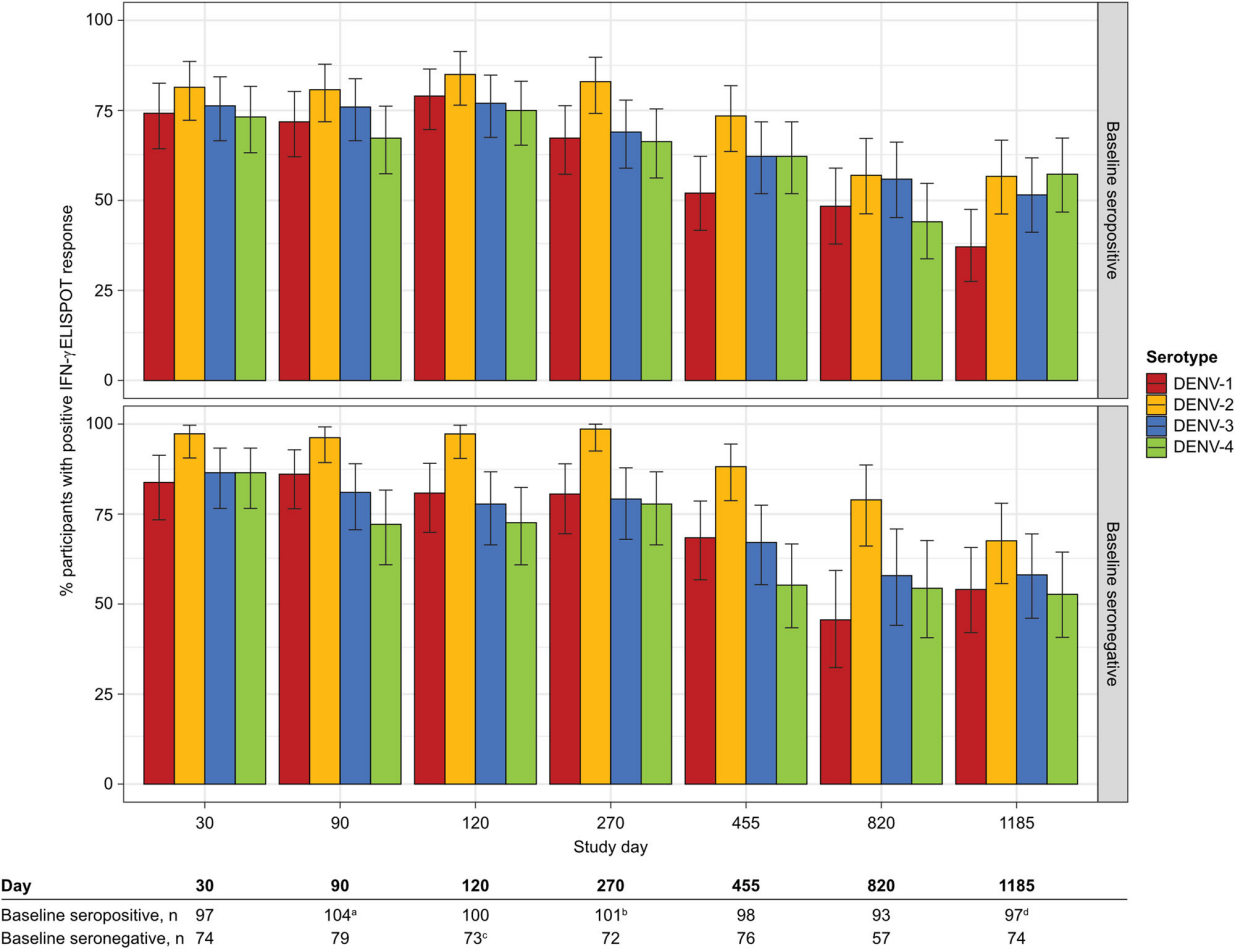
T cell response rates against individual DENV serotypes induced by TAK-003. Percentage (error bars: 95% CI) of participants with a positive T cell response to peptide pools matching DENV-1, DENV-2, DENV-3, and DENV-4 from first vaccination to Day 1185, by baseline serostatus (per protocol set). A positive T cell response was defined as IFN-γ ELISPOT response >3 times higher than baseline and ≥5 spot forming cells [SFCs]/10^6^ peripheral blood mononuclear cells [PBMCs]). LOD: 5 SFC/10^6^ PBMC. The terms “seropositive” and “seronegative” refer to baseline dengue serostatus. Seropositivity was defined as a neutralizing titer MNT ≥ 10 for at least one DENV serotype. Peptide pools evaluated: DENV-1, -2, -3, and -4 NS3, and DENV-1, -2, -3, and -4 NS5. The table indicates the number of participants with samples for each time point. ^a^*n* = 103 for DENV-1; ^b^*n* = 100 for DENV-2 and -3; ^c^*n* = 72 for DENV-3; ^d^*n* = 96 for DENV-4. ELISPOT enzyme-linked immunospot, IFN-γ interferon-gamma.

One month after the second dose, IFN-γ ELISPOT T cell response rates to peptide pools matching serotypes DENV-1, -2, -3, and -4 were 79.8%, 90.2%, 77.3%, and 74.0%, respectively, and remained elevated through the end of Year 3, irrespective of serostatus (Fig. 3).

**Fig. 4:**
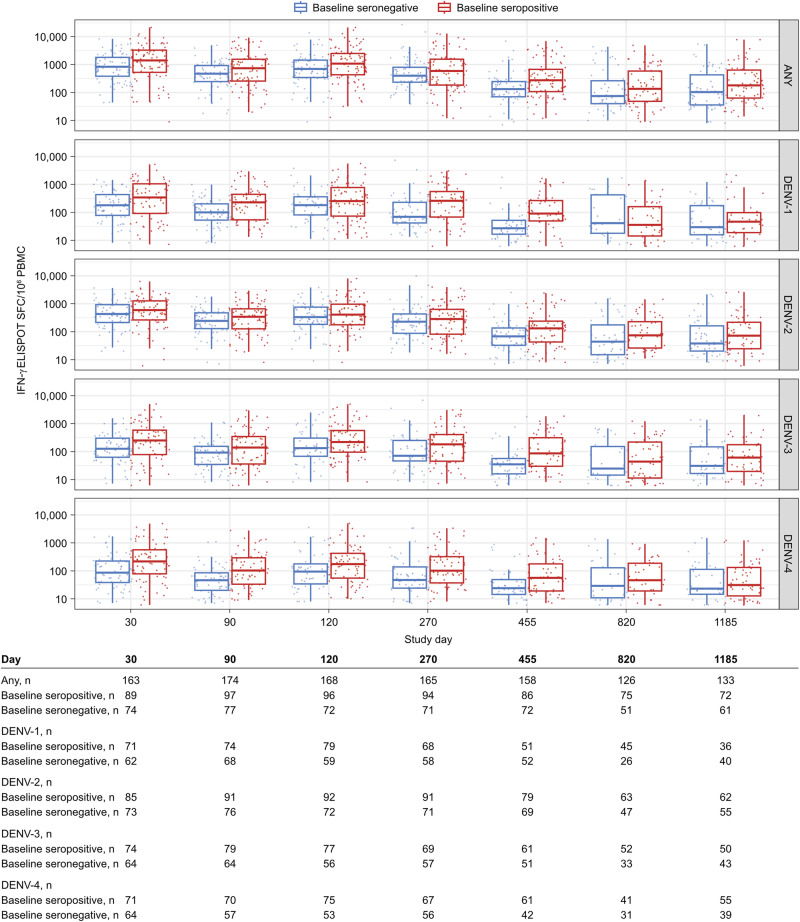
Magnitude of T cell responses against DENV induced by TAK-003. Boxplot of median magnitude (error bars: interquartile range) of T cell responses to any DENV peptide pool and peptide pools matching DENV-1, DENV-2, DENV-3, and DENV-4 in participants with a positive T cell response from first vaccination to Day 1185, by baseline serostatus (per protocol set). A positive T cell response was defined as IFN-γ ELISPOT response >3 times higher than baseline and ≥5 SFC/10^6^ peripheral blood mononuclear cells [PBMCs]. Seropositivity was defined as a neutralizing titer MNT ≥ 10 for at least one DENV serotype. Limit of detection (LOD): 5 SFC/10^6^ PBMC. Peptide pools evaluated for “any” serotype: DENV-2 NS1, DENV-1, -2, -3, and -4 NS3, and DENV-1, -2, -3, and -4 NS5. Peptide pools evaluated for individual serotypes included NS3 and NS5 only for the corresponding serotype. Magnitude estimates for individual serotypes were calculated by adding together negative control-subtracted magnitude measures against individual peptides. The table indicates the number of participants with samples for each time point. ELISPOT enzyme-linked immunospot, IFN-γ interferon-gamma, PBMC peripheral blood mononuclear cells, SFC spot-forming cells. Boxplot elements: center line, median; box limits, upper and lower quartiles; whiskers, 1.5× interquartile range; points, outliers.

The median magnitude of IFN-γ ELISPOT responses to any peptide pool and peptide pools matching serotypes DENV-1, -2, -3, and -4 post-second dose was numerically higher in seropositive participants compared to seronegative participants (e.g., any peptide pool: 1086 vs 700 Day 120, respectively); thereafter responses contracted and then stabilized through Year 3 (Fig. 4 and Supplementary Fig. 2).

**Fig. 5:**
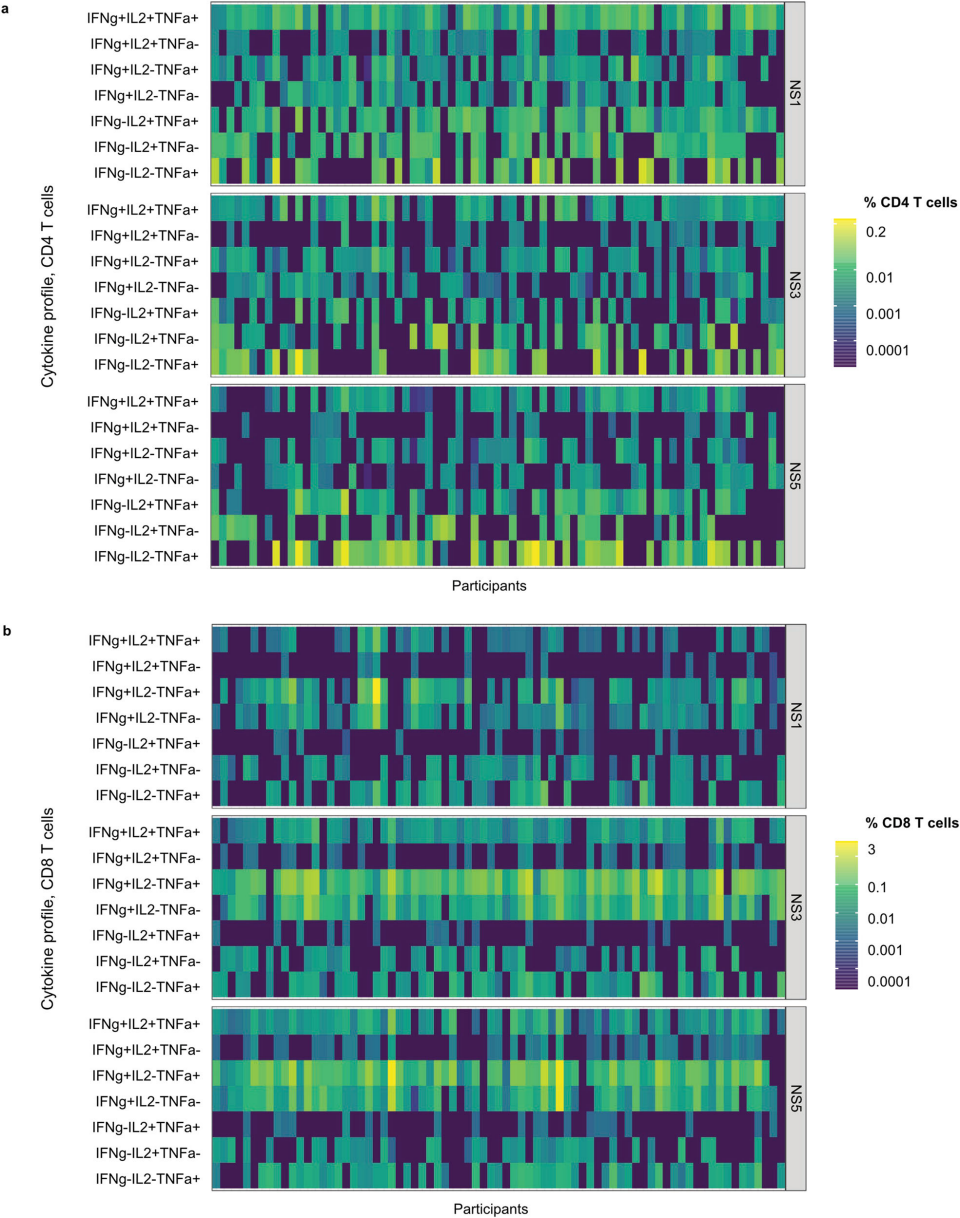
Cytokine profiles of T cell responses against DENV NS proteins induced by TAK-003. Heat maps of cytokine profiles for individual participants for **a** CD4 and **b** CD8 T cell response against DENV-2 NS1, NS3, and NS5 proteins one month after the second vaccination (Day 120) (ICS subset). The legend maps colors for individual subjects and cytokine profiles to magnitudes expressed as % cytokine-secreting CD4 or CD8 T cells. Data are shown for each of the tested peptide pools. IFNg interferon-gamma, IL2 interleukin 2, NS non-structural protein, TNFa tumor necrosis factor-alpha.

**Fig. 6:**
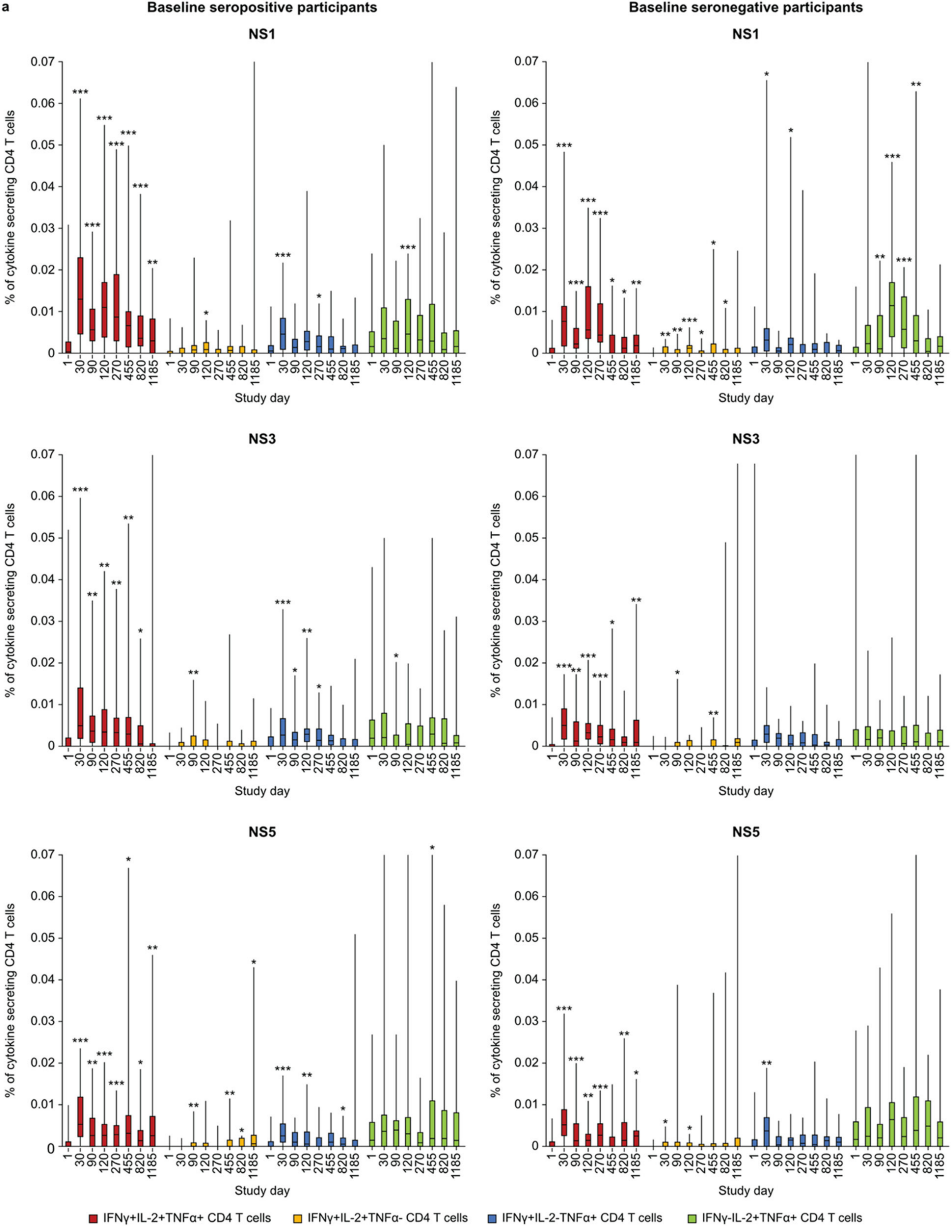

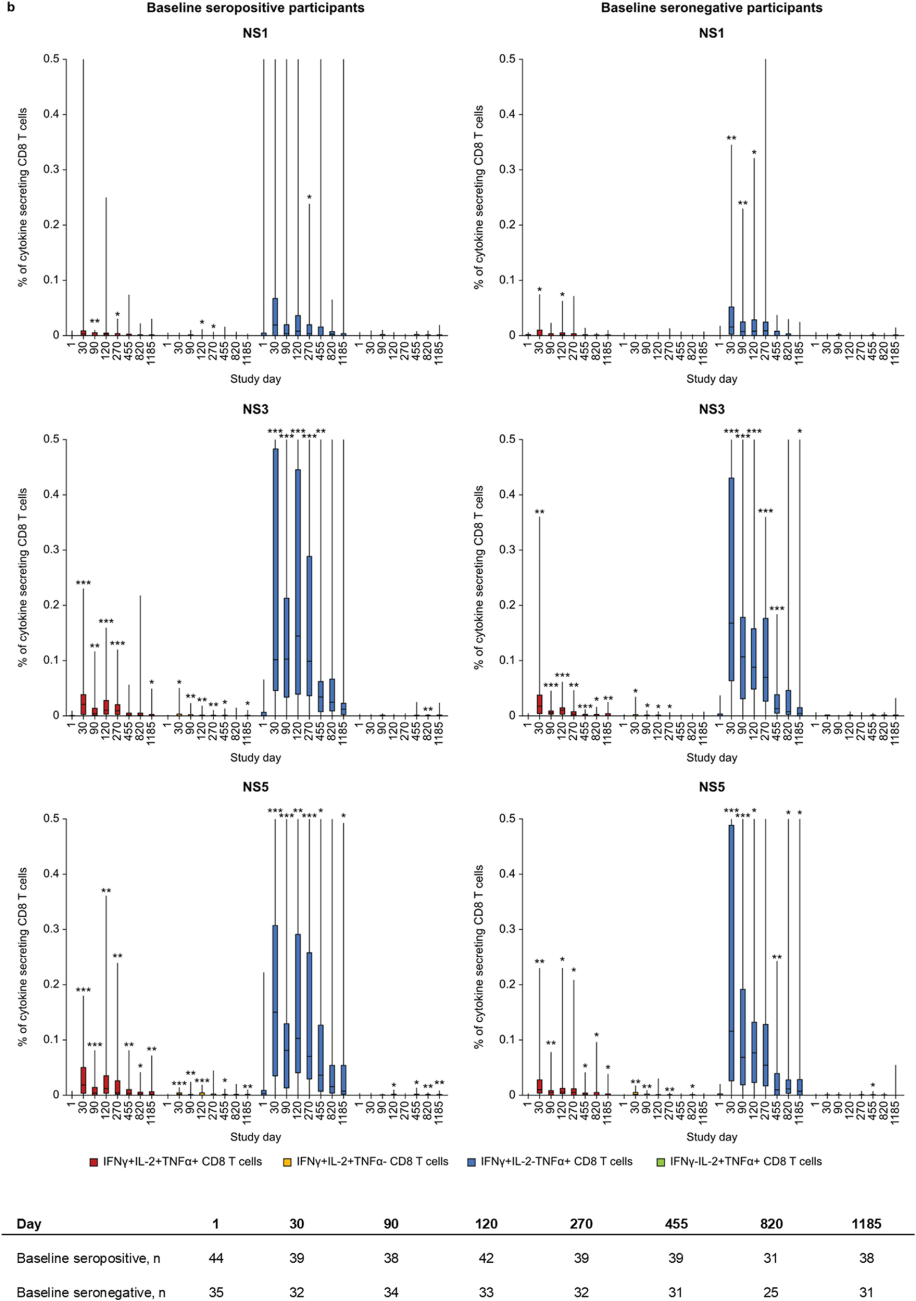
Multifunctional T cell responses against DENV NS proteins induced by TAK-003. Percentage (error bars: 95% CI) of CD4 and CD8 T cells secreting ≥2 cytokines against DENV-2 NS1, NS3, and NS5 from first vaccination to Day 1185, by baseline dengue serostatus (ICS subset). *p* Values represent the frequency of antigen-specific T cell subsets vs baseline using paired *t*-tests *p < 0.05; **p < 0.01; ***p < 0.001. Data are truncated at 0.07% for CD4 T cells and 0.5% for CD8 T cells for clarity. The terms “seropositive” and “seronegative” refer to baseline dengue serostatus. Seropositivity was defined as a neutralizing titer MNT ≥ 10 for at least one DENV serotype. The table indicates the number of participants with samples for each time point.

CD4 T cells were multifunctional and mostly characterized by IL2 + TNFα + IFNγ+ cells targeting DENV-2 NS1, NS3, and NS5 (Fig. 5) which remained increased above baseline levels up to 3 years post-vaccination, irrespective of baseline serostatus (Fig. 6a). Additionally, IFN-γ-TNFα + IL2+, IFNγ + IL-2 + TNFα-, and IFNγ + TNFα + IL-2-CD4 T cells were observed (Fig. 6a). CD8 T cell responses were typically targeting DENV-2 NS3 and NS5 and characterized by secretion of IFN-γ with or without TNF-α (Fig. 5) with smaller percentage of CD8 T cells being IFN-γ + TNFα + IL2+ (Fig. 6b). Similarly, to CD4 T cell responses, these multifunctional CD8 T cell responses were elicited in both baseline seropositive and seronegative participants, with persistent responses above baseline levels through 3 years post-vaccination.

### NAb titers and seropositivity rates

Geometric mean titers (GMTs) of NAbs increased from baseline to Day 30 (after the second dose) and remained above baseline levels throughout the study in both baseline seropositive and seronegative participants (Supplementary Fig. 3). Responses to all four DENV serotypes were observed, irrespective of baseline serostatus.

Seropositivity rates were high post-vaccination against all serotypes and remained elevated up to three years post-second dose in both baseline seropositive and seronegative participants. Tetravalent seropositivity one month after the second dose was 100% in baseline seropositive and 98.7% in baseline seronegative participants (Supplementary Fig. 4).

### Analysis of T cell and NAb responses in VCD cases

In total, 520 febrile illness cases were reported during the study, of which 12 (6%) were confirmed as VCD (Table 3). One case (DENV-1) occurred in Panama, while the other 11 cases occurred in the Philippines, with all four DENV serotypes identified across the 12 cases. Overall, 7 (58%) cases occurred in baseline seronegative participants. Participants with VCD were clinically diagnosed with dengue fever (*n* = 2), systemic viral illness (*n* = 4), urinary tract infection (*n* = 2), upper respiratory tract infection (*n* = 2), acute nasopharyngitis (*n* = 1), or acute rhinopharyngitis (*n* = 1). One participant had signs of hemorrhagic tendency (petechiae), and none of the participants showed evidence of plasma leakage in clinical assessment. None of the cases met the WHO 1997 criteria for DHF. For one participant (baseline seronegative, causative serotype: DENV-1), hospitalization was required due to dengue fever, and the event was reported as a serious AE (SAE). All events were observed after the second TAK-003 dose and resolved within 2–7 days.

**Table 3:** Summary of virologically-confirmed dengue cases (safety set)

	Panama, (*n* = 100)	Philippines, (*n* = 100)	Total, (*N* = 200)
Total number of febrile illness cases^a^	123	397	520
Participants with VCD^b^, *n* (%)	1 (1.0)	11 (11.0)	12 (6.0)
DENV-1	1 (100)	2 (18.2)	3 (25.0)
DENV-2	0 (0.0)	2 (18.2)	2 (16.7)
DENV-3	0 (0.0)	5 (45.5)	5 (41.7)
DENV-4	0 (0.0)	2 (18.2)	2 (16.7)
Baseline serostatus^c^, *n* (%)			
Seropositive	1 (100)	4 (36.4)	5 (41.7)
Seronegative	0 (0.0)	7 (63.6)	7 (58.3)

^a^Any participant with febrile illness (defined as a temperature ≥ 38 °C on two consecutive days) or clinically suspected dengue was considered for febrile illness evaluation.

^b^VCD is defined as a febrile illness with a positive DENV serotype-specific RT-PCR result.

^c^Seropositivity is defined as a neutralizing titer MNT ≥ 10 for at least one DENV serotype.

Evaluation of immune responses in TAK-003 recipients who developed VCD showed both T cell and NAb responses post-vaccination, irrespective of baseline serostatus. Overall, 9 of the 12 participants with VCD had lower IFN-γ ELISPOT T cell responses at study time points prior to developing VCD compared with median IFN-γ ELISPOT T cell responses for participants who did not have VCD (Supplementary Tables 1 and 2). Similarly, 7 of the 12 participants with VCD had lower GMTs of NAb responses compared with the median GMT of NAb responses for participants who did not have VCD (one participant was only marginally below the median) (Supplementary Tables 3 and 4). Across baseline seropositive and seronegative participants, DENV infection generally resulted in an increase in the magnitude of both T cell and NAb responses across DENV serotypes, indicating an engagement of both arms of the immune system in dengue infection (Supplementary Fig. 5). Similar trends were observed, irrespective of the causative DENV serotype (Supplementary Fig. 6).

### Safety

Overall, 42% of participants experienced unsolicited adverse events (AEs) after any vaccination, all of which were considered mild-to-moderate in severity with few reporting vaccine-related events (1.5%) (Table 4). All were non-serious. The most commonly occurring AEs by preferred term were nasopharyngitis and upper respiratory tract infection. In total, 50.5% of participants experienced medically-attended AEs (MAAEs) after any vaccination, all of which were considered mild-to-moderate in severity. The most common MAAEs by preferred term were upper respiratory tract infection, nasopharyngitis, and pneumonia. Eleven participants reported SAEs, none of which were considered related to the vaccine. Four participants had vaccine viremia after the first dose, none of which were clinically diagnosed as dengue, and two participants reported viral rash. No AEs leading to vaccine withdrawal or study discontinuation or deaths were reported (Table 4).

**Table 4:** Safety data overview (safety set)

	After first vaccination, (*N* = 200)		After second vaccination, (*N* = 198)			After any vaccination, (*N* = 200)
	Events	Participants, (%)	Events	Participants, (%)	Events	Participants, (%)
All unsolicited AEs	68	53 (26.5)	58	50 (25.3)	126	84 (42.0)
Related to IP	0	0	3	3 (1.5)	3	3 (1.5)
Related to trial procedure	0	0	0	0	0	0
Mild	65	50 (25.0)	56	49 (24.7)	121	80 (40.0)
Moderate	3	3 (1.5)	2	1 (0.5)	5	4 (2.0)
Leading to discontinuation	0	0	0	0	0	0
All MAAEs	159	84 (42.0)	202	89 (44.9)	361	101 (50.5)
Related to IP	0	0	3	3 (1.5)	3	3 (1.5)
Mild	157	82 (41.0)	202	89 (44.9)	359	99 (49.5)
Moderate	2	2 (1.0)	0	0	2	2 (1.0)
Leading to discontinuation	0	0	0	0	0	0
All SAEs	1	1 (0.5)	15	10 (5.1)	16	11 (5.5)
Related to IP	0	0	0	0	0	0
Mild	0	0	0	0	0	0
Moderate	1	1 (0.5)	14	9 (4.5)	15	10 (5.0)
Severe	0	0	1	1 (0.5)	1	1 (0.5)
Leading to discontinuation	0	0	0	0	0	0
Deaths	0	0	0	0	0	0
VCD	0	0	12	12 (6.0)	12	12 (6.0)

*AE* adverse event, *IP* investigational product, *MAAE* medically-attended adverse event, *SAE* serious adverse event, *VCD* virologically confirmed dengue.

## Discussion

In this study, vaccination of children and adolescents with TAK-003 resulted in durable cellular and humoral immune responses which persisted above baseline levels for at least 3 years, independent of baseline serostatus. Overall, the results of this study complement the efficacy, safety, and immunogenicity findings from the phase 3 efficacy study in children aged 4–16 years living in areas of Asia and Latin America considered endemic for dengue^18–22^, and provide insight into how TAK-003 elicits immune responses in recipients across previous exposure backgrounds, ages, and regions.

While evaluation of vaccine immunogenicity often focuses predominantly on antibody responses, cellular immunity plays a key role in protection from many diseases, including dengue^9–14,27–29^. In both dengue-naïve and previously-exposed participants, TAK-003 elicited a strong and rapid T cell response, with over 90% of subjects achieving a positive response within a month of receiving the first dose. Responses were seen across all four serotypes. Subgroup analysis in participants > 10 years of age showed that T cell responses were evident within 14 days of vaccination, however, further evaluation in larger numbers of participants is needed to assess any potential serostatus differences. Irrespective of baseline dengue serostatus, IFN-γ ELISPOT T cell responses to all four DENV serotypes persisted through three years post-vaccination.

Multifunctional DENV-specific T cells are associated with a protective immune response against natural DENV infection^14,24,25,27,30^. While one limitation of the study was that we only performed ICS using DENV-2 peptides, we observed CD4 T cell responses typically involving triple cytokine-secreting cells targeting DENV-2 NS1, NS3, and NS5, whereas the CD8 T cell response predominantly involved secretion of IFN-γ and TNF-α targeting DENV-2 NS3 and NS5, with responses maintained through Year 3, irrespective of the dengue baseline serostatus. As expected, the frequencies of DENV‑2 NS3 and NS5-specific CD4 T cells were noticeably lower than observed for the CD8 T cells, suggesting that the cellular immune response may be predominantly mediated by CD8 T cells, which is consistent with findings from the previous phase 2 study in adolescents^25^.

The findings of this study are in contrast to the analysis of T cell responses to the only other licensed dengue vaccine, CYD-TDV. Unlike TAK-003, which uses a DENV-2 backbone, CYD-TDV is based on a yellow fever YF-17D backbone. An initial evaluation of T cell response following receipt of CYD-TDV showed significant CD8 T cell responses targeting the YF-17D backbone, with CD4 T cell responses directed against the DENV envelope protein^31^. In dengue-naïve participants, T cell responses after initial CYD-TDV vaccination were focused mainly against DENV-4, although the response became broader across all four serotypes after subsequent doses^31–33^. In contrast, we observed T cell responses to all four DENV serotypes irrespective of serostatus after the first dose of TAK-003, with 37.1%, 56.7%, 51.6%, and 57.3% of baseline seropositive and 54.1%, 67.6%, 58.1%, and 52.7% of baseline seronegative participants still showing responses to DENV-1, -2, -3, or -4, respectively, three years post-vaccination. In contrast to the differences from CYD-TDV, TAK-003 demonstrated a very similar T cell response profile to the TV003 investigational dengue vaccine, which contains elements encoding NS proteins for DENV-1, -3, and -4^34,35^. Although the TAK-003 backbone includes only DENV-2 specific NS1, NS3, and NS5 proteins, conservation of these proteins across DENV serotypes likely resulted in simultaneous induction of T cell responses against DENV-1, -3 and -4 following vaccination with TAK-003. This durable, multitypic T cell immunity may contribute to the prevention of severe dengue in TAK-003 vaccinees^22^ and is consistent with the vaccine’s long-term efficacy and safety profile, where no increase in severity of disease has been observed in vaccinees with breakthrough infections along with durable high level of protection against dengue leading to hospitalization^18–22,36,37^.

Overall, the immunogenicity and safety profile in this study were similar to those reported throughout the TAK-003 clinical development program, with no new safety risks identified^18–22,36^. Analysis of the small number of breakthrough VCD cases showed expansion of T cells and NAb responses against all four DENV serotypes upon infection, irrespective of the causative serotype. In natural infections, strong T cell responses have been associated with the prevention of severe dengue^14^, and in line with this, nearly all the VCD cases in the current study were mild. In addition, repeat infections with different dengue serotypes are believed to induce distinct immune response profiles compared with primary infections, which is believed to be the basis for the mild symptoms associated with tertiary and later infections^38^. We hypothesize that individuals with breakthrough infections post-vaccination with TAK-003 may still derive indirect benefit of vaccination against future infections. In the pivotal efficacy study DEN-301, the risk of sequential symptomatic dengue infections was lower in TAK-003 recipients in comparison to placebo controls^39^.

One of the major strengths of this study was that it was specifically designed to evaluate T cell responses, with a long-term follow-up period. Additionally, the study was designed to be similar to the setting and participant characteristics of the large-scale pivotal phase 3 efficacy study^22^, to maximize the clinical applicability of the findings. As there is no consensus on the definition of a positive T cell response, we used a definition that allowed adjustment for baseline levels in order to focus solely on the vaccine-induced response. The occurrence of breakthrough cases also allowed us to investigate responses to natural infection following vaccination, although data are limited due to the small number of cases reported. One limitation of the study was the lack of a placebo arm. We chose not to include placebo in light of the expected minimal variation in responses over time in the absence of vaccination or natural exposure and significant inter-individual variability of baseline responses. Therefore, the study was designed as an open-label study, with all participants receiving the vaccine, with baseline T cell responses serving as the reference for evaluation of vaccine effect.

In summary, the results of this study show that TAK-003 elicited persistent T cell responses against all four DENV serotypes, irrespective of baseline serostatus in children and adolescents aged 4–16 years. Responses were rapid and durable through 3 years post-vaccination. TAK-003-elicited CD4 and CD8 T cells were multifunctional and persisted up to 3 years post-vaccination. The results of this study complement the efficacy findings from the phase 3 study in children and adolescents living in dengue-endemic regions, where long-term efficacy was observed against symptomatic and hospitalized dengue up to 54 months after the second vaccine dose. Overall, these data provide insight into the cellular and humoral immune responses elicited by TAK-003, as a potential mechanistic basis of vaccine protection.

## Methods

### Study overview and participants

This open-label, phase 2 study was performed at two sites in Panama (one site) and the Philippines (one site) between April 2017 and December 2020. The study was conducted in accordance with the International Council for Harmonization of Technical Requirements for Registration of Pharmaceuticals for Human Use and Good Clinical Practice (ICH E6(R2)-GCP) principles that have their origins in the Declaration of Helsinki, and applicable regulatory requirements. The protocol and informed consent forms (ICFs) were reviewed and approved by the appropriate ethics committees (Research Institute for Tropical Medicine Institutional Review Board and Comité de Bioética en Investigación del Hospital del Niño) prior to the inclusion of any participants in the study. Written informed consent/assent was obtained from all parents or legal guardians and the participants prior to enrollment. Participants who turned 18 during the study were required to give informed consent to remain in the study. The study was registered at clinicaltrials.gov (NCT02948829).

Healthy children aged 4–16 years in Panama or 4–8 years in the Philippines were eligible for enrollment. Age ranges were chosen to reflect those eligible for participation in the phase 3 efficacy study, however, enrollment in the Philippines was limited to 4–8 years-old to increase the chances of enrolling dengue seronegative individuals.

### Inclusion and exclusion criteria

Eligibility was determined according to the following criteria: 1. the participant was aged 4–16 years, inclusive (Latin America) or 4–8 years, inclusive (Asia); 2. individuals who were in good health at the time of entry into the trial as determined by medical history, physical examination (including vital signs), and clinical judgment of the investigator; 3. the participant or participant’s parent(s)/legally authorized representative signed and dated a written ICF and any required privacy authorization prior to the initiation of any trial procedures, after the nature of the trial had been explained according to local regulatory requirements; and 4. individuals could comply with trial procedures and were available for the duration of follow-up.

Any individual who met any of the following criteria did not qualify for entry into the trial: 1. febrile illness (body temperature ≥38 °C) or moderate or severe acute illness or infection at the time of enrollment; 2. history or any illness that, in the opinion of the investigator, could interfere with the results of the trial or pose an additional risk to the subject due to participation in the trial, including but not limited to: (a) known hypersensitivity or allergy to any of the vaccine components, (b) female subjects (post-menarche) who were pregnant or breastfeeding, (c) individuals with any serious chronic or progressive disease (eg, neoplasm, insulin-dependent diabetes, cardiac, renal or hepatic disease, neurologic or seizure disorder or Guillain-Barré syndrome), (d) known or suspected impairment/alteration of immune function, including: i. chronic use of oral steroids (equivalent to 20 mg/day prednisone ≥ 12 weeks/≥2 mg/kg body weight/day prednisone ≥ 2 weeks) within 60 days prior to Day 1 (Month 0 [M0]0) (use of inhaled, intranasal, or topical corticosteroids is allowed), ii. receipt of parenteral steroids (equivalent to 20 mg/day prednisone ≥ 12 weeks/≥2 mg/kg body weight/day prednisone ≥ 2 weeks) within 60 days prior to Day 1 (M0), iii. administration of immunoglobulins and/or any blood products within the 3 months prior to Day 1 (M0) or planned administration during the trial, iv. receipt of immunostimulants within 60 days prior to Day 1 (M0), v. immunosuppressive therapy such as anti-cancer chemotherapy or radiation therapy within 6 months prior to Day 1 (M0), vi. HIV infection or HIV-related disease, and vii. genetic immunodeficiency. 3. receipt of any other vaccines within 14 days (for inactivated vaccines) or 28 days (for live vaccines) prior to Day 1 (M0) or planning to receive any vaccines within 28 days after Day 1 (M0); 4. participation in any clinical trial with another investigational product 30 days prior to Day 1 (M0) or intent to participate in another clinical trial at any time during the conduct of this trial; 5. previous participation in any clinical trial of a dengue candidate vaccine, or previous receipt of any dengue vaccines (investigational or licensed); 6. first-degree relatives of individuals involved in the conduct of the trial. 7. females of childbearing potential who were sexually active, and who had not used any of the acceptable contraceptive methods for at least 2 months prior to Day 1 (M0): (a) of childbearing potential was defined as status post onset of menarche and not meeting any of the following conditions: bilateral tubal ligation (at least 1 year previously), bilateral oophorectomy (at least 1 year previously) or hysterectomy, (b) acceptable birth control methods were defined as 1 or more of the following: i. hormonal contraceptive (such as oral, injection, transdermal patch, implant, cervical ring), ii. barrier (condom with spermicide or diaphragm with spermicide) each and every time during intercourse, iii. intrauterine device, and iv. monogamous relationship with a vasectomized partner (partner must have been vasectomized for at least 6 months prior to Day 1 [M0]), other contraceptive methods could have been considered in agreement with the sponsor if approved by the appropriate ethics committee; 8. females of childbearing potential who were sexually active, and who refused to use an acceptable contraceptive method or avoid donation of ova up to 6 weeks post second vaccination; 9. deprived of freedom by administrative or court order, or in an emergency setting, or hospitalized involuntarily; 10. current alcohol abuse or drug addiction that could have interfered with the individual’s ability to comply with trial procedures; and 11. identified as an employee of the investigator or trial center, with direct involvement in the proposed trial or other trials under the direction of that investigator or trial center.

There could have been instances when individuals met all entry criteria except one that related to transient clinical circumstances (e.g., body temperature elevation or recent use of excluded medication or vaccine). Under these circumstances, eligibility for first vaccination could have been considered if the appropriate window for delay had been passed, inclusion/exclusion criteria had been rechecked, and if the subject was confirmed to be eligible.

Participants who were otherwise eligible, but could not receive the trial vaccination because the limit of approximately 60% of subjects (i.e., sample size at each trial center) with the same dengue baseline seropositivity status was reached, did not continue in the trial and became screen failures.

### Study design

All participants received a two-dose schedule of TAK-003, administered as a subcutaneous injection into the upper arm on Days 1 and 90. Enrollment was stratified to ensure a balance (≥40% each) of dengue seropositivity at each site at baseline.

Blood samples were taken for assessment of baseline serostatus at screening using a dengue IgG indirect ELISA (Panbio dengue indirect IgG for The Philippines and Focus Diagnostics dengue virus IgG DxSelect for Panama). Blood samples were also taken on Days 1, 14 (a subgroup of participants > 10 years only; see below), 30, 90, 120, and 270, and then annually for 3 years post-second dose for evaluation of T cell responses and NAb titers (Supplementary Fig. 7). Seropositivity was defined using a validated microneutralization test (MNT) as neutralizing titer ≥ 10 against at least one dengue serotype; participants were seronegative if they were not seropositive against any of the four dengue serotypes at baseline. The additional blood sample on Day 14 of the study was performed to evaluate early onset T cell responses but was limited to participants > 10 years of age due to the constraints of blood volume collection in children ≤ 10 years of age^40^.

### Vaccine

The vaccine used in this study was of the same potency as the vaccine used in the phase 3 efficacy study. A single 0.5 mL dose of the vaccine contained 3.6 log_10_ pfu of TDV-1, 4.0 log_10_ pfu of TDV-2, 4.6 log_10_ pfu of TDV-3, and 5.1 log_10_ pfu of TDV-4.

### NAb and T-cell assays

NAbs in serum samples were evaluated using a validated MNT, with results expressed as the reciprocal of the highest dilution of test serum that shows a 50% reduction in plaque counts compared with that of virus controls. Peripheral blood mononuclear cells (PBMCs) were used to evaluate T cell responses using an IFN-γ enzyme-linked immunospot (ELISPOT) assay (Mabtech AB, catalog#3420) with positive samples being further tested using flow cytometry-based ICS assay (ICS subset; see “Statistical analysis” section below). For the ELISPOT assay, plates were coated with 100 µL/well anti-IFN-γ antibody solution and incubated overnight at 6 °C ± 2 °C. Plates were then washed and 100 µL antigen mix was added to each well. DENV-specific peptide pools were reconstituted in dimethyl sulfoxide, diluted in cell culture medium, and used at a final concentration of 1 µg/mL/peptide. Anti-CD3 antibody (Mabtech, catalog#3605-1) was used as a positive control, and medium plus 0.5% dimethyl sulfoxide was used as a negative control. After thawing and overnight resting, PBMCs were resuspended at 1.0 to 2.0 × 10^6^ cells/mL, and 100 µL added to each well. Plates were then incubated at 37 °C, 5% atmosphere CO_2_ overnight. An automated ELISPOT counter (CTL S6 Universal-V analyzer) was used to count the number of spot-forming cells (SFC) per well, which were normalized to SFC/10^6^ PBMC for final interpretation. For ICS evaluation, cryopreserved PBMCs were thawed and rested overnight. Cells were then washed and cultured together with 1 µg/mL/peptide DENV-specific peptide pools (DENV-2 NS1, NS3, and NS5) at 37 °C for 1 h. Protein transport inhibitors (Golgi Plug/Stop, BD Biosciences, #555029 and #554724) were added and the incubation continued for an additional 6 hours. Cells were stained for viability and with the following antibody panel: CD3 (dilution 1:160, BV785, Biolegend, catalog#317330), CD4 (1:80, BV605, Biolegend, #300556), CD8 (1:160, BV650, Biolegend, #301042), IFN-γ (1:20, eF450, eBioscience, #48-7319-42), TNF-α (1:160, eBioscience, #25-7349-82), IL-2 (1:40, BD Pharmingen, #554567), CD14 (1:160, A700, BD Pharmingen, #557923), and CD19 (1:160, A700, BD Pharmingen, #557921). Data were acquired on a BD LSRFortessa flow cytometer and analyzed with FlowJo v10 software (Supplementary Fig. 8).

For the primary analysis, the frequency of responses to any tested peptide pool (NS1 for DENV-2, and NS3 and NS5 for DENV-1, -2, -3, and -4) was evaluated. Secondary analysis was also performed on peptide pools matching a given dengue serotype (NS3 and NS5 only), where response to at least one serotype-specific peptide pool was classed as positive. ICS analysis was performed on responses to DENV-2 peptides (NS1, NS3, and NS5). A positive T cell response was defined as an IFN-γ ELISPOT response > 3 times higher than baseline (i.e., day 1) and ≥5 SFC/10^6^ PMBCs. ELISPOT and ICS responses underwent a background correction which involved subtracting the response of the negative control from each value. Magnitude estimates for individual serotypes were calculated by adding together negative control-subtracted magnitude measures against individual peptides.

### Study objectives

The primary objective of the study was to assess the IFN-γ ELISPOT T cell response to two doses of TAK-003 at one month after the second vaccination (Day 120). Secondary objectives included IFN-γ ELISPOT T cell responses from one month up to 3 years post-second vaccination, phenotypic characterization of T cell responses by ICS up to 3 years post-second vaccination, evaluation of NAb titers, seropositivity rates, and safety. Post-hoc analysis included evaluation of T cell and NAb responses in participants who experienced virologically-confirmed dengue (VCD), defined as febrile illness with a positive serotype-specific RT-PCR, post-vaccination. Participants were contacted at least weekly to ensure robust identification of any febrile illness, and blood samples were taken from any participant with suspected dengue or fever ≥38 °C for 2 of 3 consecutive days to confirm dengue infection. Safety was evaluated in terms of unsolicited AEs up to 28 days after each vaccination; MAAEs up to 6 months post-second dose; and SAEs, AEs leading to vaccine withdrawal or study discontinuation, and febrile episodes with potential dengue etiology up to the end date of the study.

### Statistical analysis

No formal statistical hypotheses were tested in this study; data are presented as descriptive statistics. IFN-γ ELISPOT T cell responses and immunogenicity data, with the exception of ICS, are presented for the PPS, i.e., all participants who received at least one dose of the study vaccine and for whom a valid pre-dosing and at least one post-dosing blood sample were received, and had no major protocol violations. ICS results are presented for the ICS subset, i.e., participants from PPS who had IFN-γ ELISPOT responses > 50 SFC/10^6^ PBMCs and availability of sufficient cells for analysis. Safety data are presented for the safety set, i.e., all participants who received at least one dose of the study vaccine. All analyses were performed using SAS version 9.4 and R version 4.3.1.

## Supplementary information

Supplementary Materials

## Data Availability

The datasets, including the redacted study protocol, redacted statistical analysis plan, and individual participants’ data supporting the results of the completed study, will be made available within three months from the initial request, to researchers who provide a methodologically sound proposal. The data will be provided after its de-identification, in compliance with applicable privacy laws, data protection, and requirements for consent and anonymization. Data requests should follow the process described in the Data Sharing section on https://clinicaltrials.takeda.com/ and https://vivli.org/ourmember/takeda/.
