# Supplementary material for: Long term T cell response and safety of a tetravalent dengue vaccine in healthy children
Source: NPJ Vaccines. 2024 Oct 17;9:192. doi: 10.1038/s41541-024-00967-0 (PMC11487277; doi:10.1038/s41541-024-00967-0)

Supplementary Table 1. Summary statistics of the magnitude of IFN-γ ELISPOT responses to any DENV peptide pool from baseline to Day 1185 across participants who did not have VCD, by baseline serostatus (per protocol set)

Day	Median	Mean	Q25	Q75	n
Baseline seropositive					
1	36	265	9	141	102
30	1251	2483	396	3257	95
90	670	1392	257	1553	101
120	1051	2308	388	2512	96
270	592	1515	190	1567	98
455	233	707	73	547	96
820	89	370	30	279	88
1185	87	490	25	268	94
Baseline seronegative					
1	12	23	0	35	74
30	885	1404	396	1844	73
90	473	650	229	916	77
120	723	1350	341	1417	70
270	390	1225	210	802	69
455	121	321	62	245	75
820	56	368	25	138	54
1185	40	255	23	123	71

ELISPOT, enzyme-linked immunospot; IFN-γ, interferon gamma, Q25, lower quartile; Q75, upper quartile

Data are presented as spot forming cells (SFC)/10^6 peripheral blood mononuclear cells (PBMC). Limit of detection: 5 SFC/10^6 PBMC. Peptide pools evaluated for “any” serotype: DENV-2 NS1, DENV-1, -2, -3, and -4 NS3, and DENV-1, -2, -3, and -4 NS5. The terms “seropositive” and “seronegative” refer to baseline dengue serostatus. Seropositivity was defined as a neutralizing titer MNT ≥10 for at least one DENV serotype.

Supplementary Table 2. Summary statistics of the magnitude of IFN-γ ELISPOT responses to any DENV peptide pool from baseline to Day 1185 for individual VCD cases, by baseline serostatus (per protocol set)

Case	VCD details	IFN-γ ELISPOT response at visit prior to VCD (any peptide pool)	Positive response pre-VCD?	IFN-γ ELISPOT response at visit post-VCD (any peptide pool)	Positive response post-VCD?
Baseline seropositive					
1	DENV-1 at Day 211	182 (Day 90)	Yes	8202 (Day 270)	Yes
2	DENV-3 at Day 353	107 (Day 270)	No	1276 (Day 820)	Yes
3	DENV-3 at Day 346	183 (Day 270)	Yes	2430 (Day 455)	Yes
4	DENV-2 at Day 738	85 (Day 455)	No	1158 (Day 820)	Yes
5	DENV-2 at Day 784	43 (Day 455)	No	0 (Day 820)	No
Baseline seronegative					
1	DENV-1 at Day 744	698 (Day 270)	Yes	1145 (Day 820)	Yes
2	DENV-3 at Day 777	1844 (Day 455)	Yes	647 (Day 820)	Yes
3	DENV-3 at Day 892	50 (Day 820)	No	1013 (Day 1185)	Yes
4	DENV-1 at Day 827	5 (Day 820)	No	717 (Day 1185)	Yes
5	DENV-3 at Day 686	55 (Day 455)	No	3032 (Day 820)	Yes
6	DENV-4 at Day 180	293 (Day 120)	Yes	400 (Day 270)	Yes
7	DENV-4 at Day 169	1003 (Day 120)	Yes	516 (Day 270)	Yes

VCD, virologically confirmed dengue

Data are presented as spot forming cells (SFC)/10^6 peripheral blood mononuclear cells (PBMC). Limit of detection: 5 SFC/10^6 PBMC. Peptide pools evaluated for "any" serotype: DENV-2 NS1, DENV-1, -2, -3, and -4 NS3, and DENV-1, -2, -3, and -4 NS5. The terms "seropositive" and "seronegative" refer to baseline dengue serostatus. Seropositivity was defined as a neutralizing titer MNT ≥10 for at least one DENV serotype.

Supplementary Table 3. Summary statistics of neutralizing antibody (MNT50) responses to any DENV serotype from baseline to Day 1185 across participants who did not have VCD, by baseline serostatus (per protocol set)

Day	Median	Mean	Q25	Q75	n
Baseline seropositive					
1	208	709	65	625	104
30	1933	4280	639	4848	98
90	1333	2953	491	4596	104
120	1672	4049	649	4211	99
270	903	2289	342	2767	102
455	564	1517	262	2078	103
820	483	1553	170	1517	88
1185	526	855	165	1217	97
Baseline seronegative					
1	0	0	0	0	79
30	268	464	171	375	76
90	133	378	100	245	79
120	219	396	163	348	71
270	101	401	78	156	73
455	82	151	66	112	77
820	62	840	51	128	54
1185	62	251	48	124	71

MNT, microneutralization titer; Q25, lower quartile; Q75, upper quartile; VCD, virologically confirmed dengue

Data are presented as geometric mean neutralizing antibody titers (MNT). Seropositivity was defined as MNT neutralizing titer ≥10 against at least one dengue serotype. Limit of detection: MNT=10 for NAb responses.

Supplementary Table 4. Summary statistics of neutralizing antibody (MNT50) responses to any DENV serotype from baseline to Day 1185 for individual VCD cases, by baseline serostatus (per protocol set)

Case	VCD details	MNT prior to VCD	MNT post-VCD
**Baseline seropositive**			
1	DENV-1 at Day 211	1609 (Day 90)	33066 (Day 270)
2	DENV-3 at Day 353	382 (Day 270)	56980 (Day 820)
3	DENV-3 at Day 346	106 (Day 270)	11246 (Day 455)
4	DENV-2 at Day 738	824 (Day 455)	49420 (Day 820)
5	DENV-2 at Day 784	784 (Day 455)	596 (Day 820)
Baseline seronegative			
1	DENV-1 at Day 744	64 (Day 455)	29778 (Day 820)
2	DENV-3 at Day 777	90 (Day 455)	69 (Day 820)
3	DENV-3 at Day 892	50 (Day 820)	1013 (Day 1185)
4	DENV-1 at Day 827	60 (Day 820)	1044 (Day 1185)
5	DENV-3 at Day 686	61 (Day 455)	6102 (Day 820)
6	DENV-4 at Day 180	168 (Day 120)	2023 (Day 270)
7	DENV-4 at Day 169	527 (Day 120)	1088 (Day 270)

MNT, microneutalization titer; VCD, virologically confirmed dengue

Data are presented as geometric mean neutralizing antibody titers (MNT). Seropositivity was defined as MNT neutralizing titer ≥10 against at least one dengue serotype. Limit of detection: MNT=10 for NAb responses.

Supplementary Figure 1. Percentage (error bars: 95% confidence interval) of participants with a positive T cell response to any DENV peptide pool from first vaccination to Day 1185 by country and age group (per protocol set)

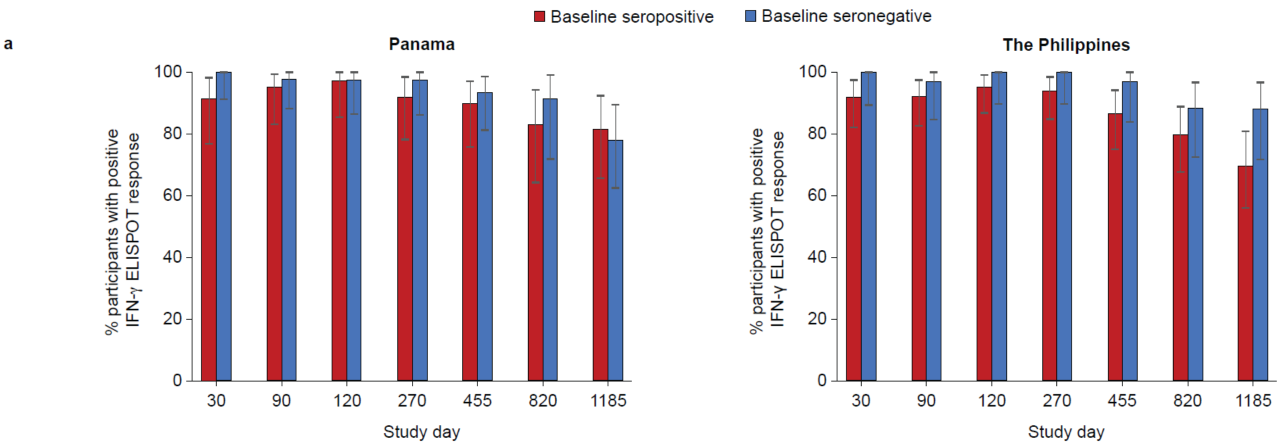

Day	30	90	120	270	455	820	1185
Baseline seropositive, n	35	40	36	37	39	29	38
Baseline seronegative, n	41	45	39	38	44	23	41

Day	30	90	120	270	455	820	1185
Baseline seropositive, n	62	64	64	64	59	64	59
Baseline seronegative, n	33	34	34	34	32	34	33

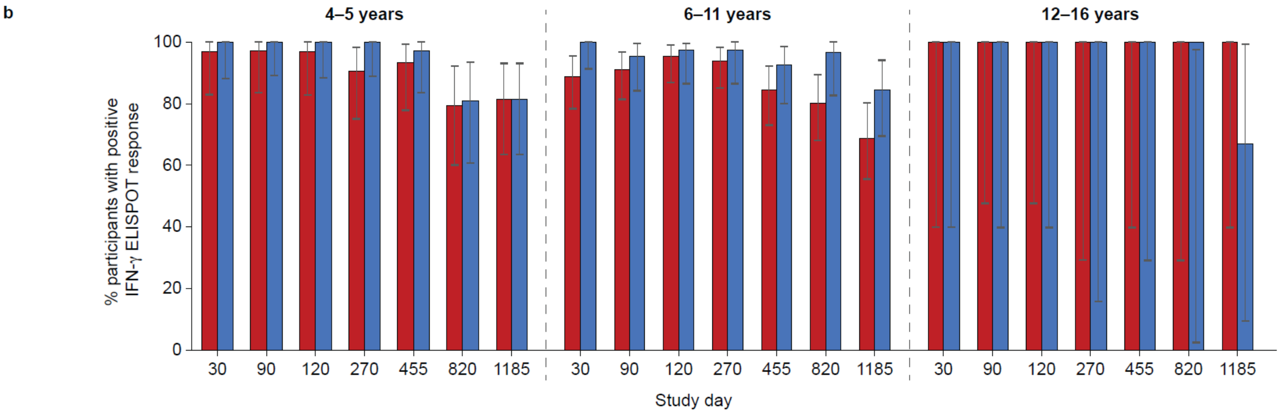

Day	30	90	120	270	455	820	1185	30	90	120	270	455	820	1185	30	90	120	270	455	820	1185
Baseline seropositive, n	30	32	30	32	30	29	32	63	67	65	66	64	61	61	4	5	5	3	4	3	4
Baseline seronegative, n	29	32	30	31	32	26	32	41	43	39	39	41	30	39	4	4	4	2	3	1	3

ELISPOT, enzyme-linked immunospot; IFN-γ, interferon-gamma.

Tables represent the number of participants included for each study visit for baseline seropositive (+) and seronegative (-) participants
A positive response was defined as IFN-γ ELISPOT response >3 times higher than baseline and ≥5 spot forming cells [SFC]/10^6 peripheral blood mononuclear cells [PBMCs]. The terms "seropositive" and "seronegative" refer to baseline dengue serostatus. Seropositivity was defined as a neutralizing titer MNT ≥10 for at least one DENV serotype. Limit of detection (LOD): 5 SFC/10^6 PBMC. Peptide pools evaluated: DENV-2 NS1; DENV-1, -2, -3, and -4 NS3, and DENV-1, -2, -3, and -4 NS5.

Supplementary Figure 2. Boxplot of median magnitude (error bars: interquartile range) of T cell responses to any peptide pool and peptide pools matching DENV-1, DENV-2, DENV-3, and DENV-4 from first vaccination to Day 1185, by baseline serostatus (per protocol set).

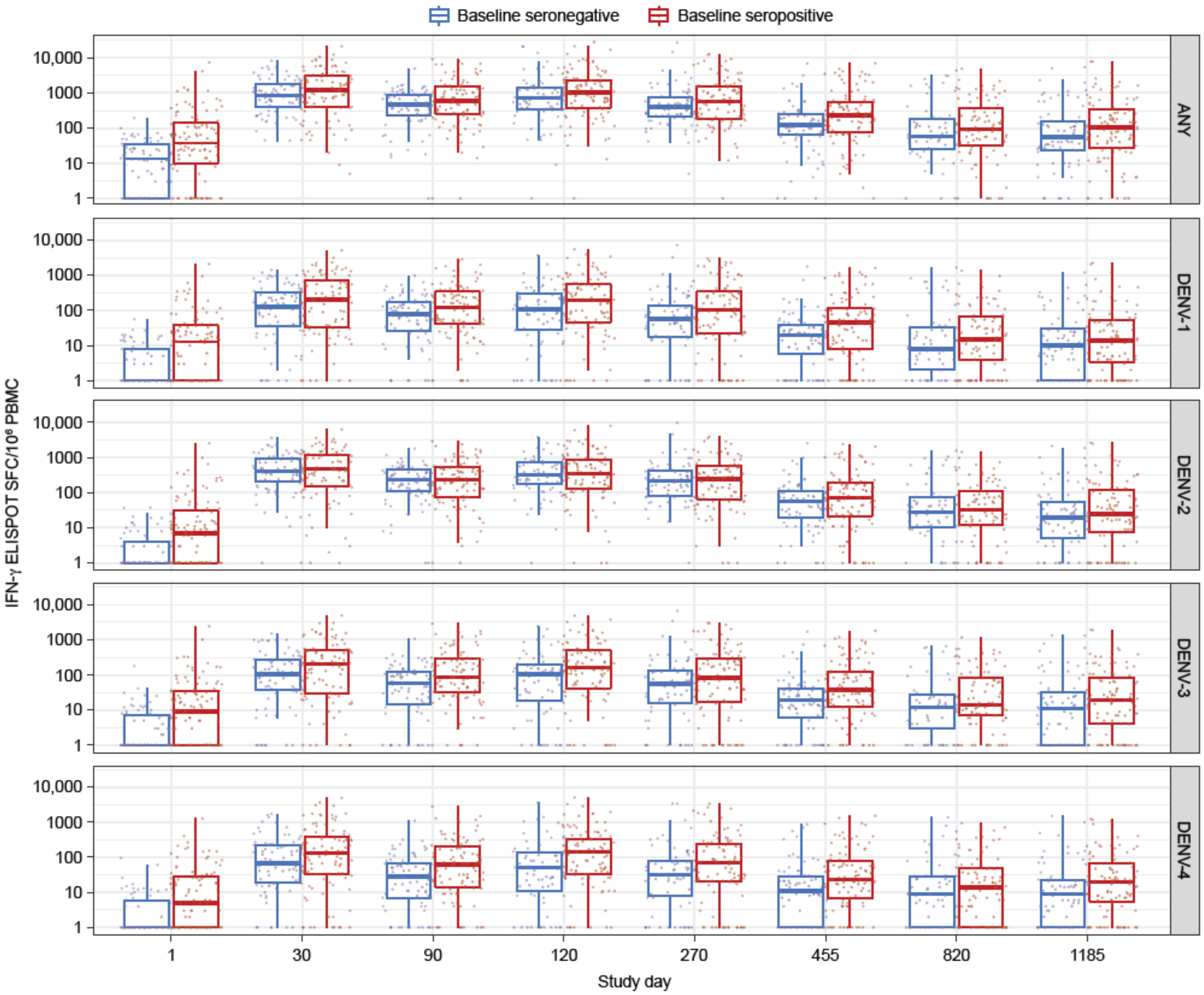

Day	1	30	90	120	270	455	820	1185
Baseline seropositive, n	107	98	106	100	103	100	93	99
Baseline seronegative, n	81	79	84	77	76	81	61	78

ELISPOT, enzyme-linked immunospot; IFN-γ, interferon-gamma; PBMC, peripheral blood mononuclear cells; SFC, spot forming cells. Boxplot elements: center line, median; box limits, upper and lower quartiles; whiskers, 1.5x interquartile range; points, outliers. The terms “seropositive” and “seronegative” refer to baseline dengue serostatus. Seropositivity was defined as a neutralizing titer MNT ≥10 for at least one DENV serotype. Limit of detection (LOD): 5 SFC/10^6 PBMC. Peptide pools evaluated for “any” serotype: DENV-2 NS1, DENV-1, -2, -3, and -4 NS3, and DENV-1, -2, -3, and -4 NS5. Peptide pools evaluated for individual serotypes included NS3 and NS5 only for the corresponding serotype. Magnitude estimates for individual serotypes were calculated by adding together negative control-subtracted magnitude measures against individual peptides. Table indicates the number of participants with samples for each time point

Supplementary Figure 3. Geometric mean titers (error bars: 95% confidence intervals) of neutralizing antibodies against individual dengue serotypes during the study (per protocol set)

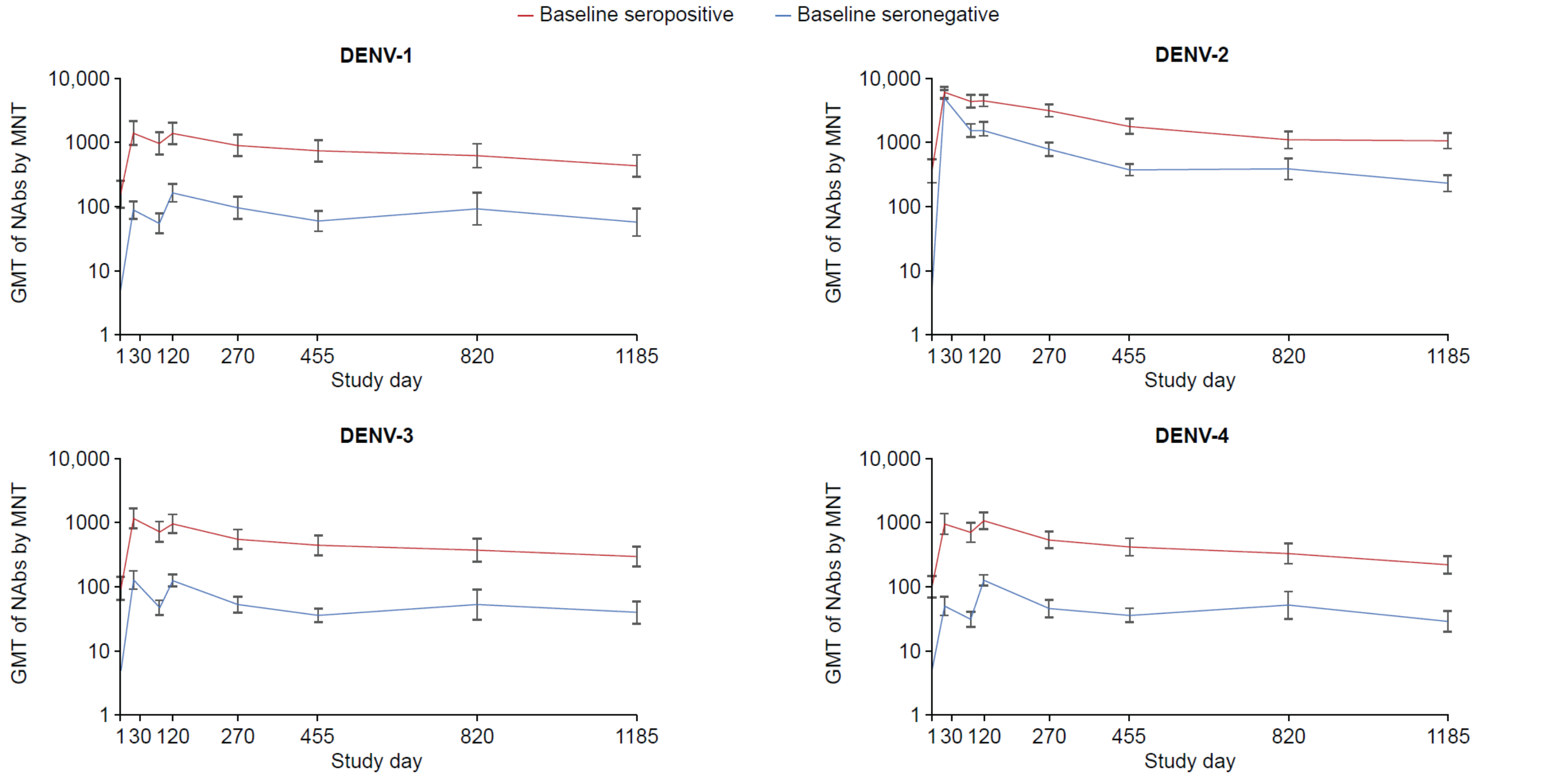

Day	1	30	90	120	270	455	820	1185
Baseline seropositive, n	109	102	109	104	107	108	93	102
Baseline seronegative, n	86	83	86	78	80	84	61	78

GMT, geometric mean titer; MNT, microneutralization test; NAb, neutralizing antibody

The terms “seropositive” and “seronegative” refer to baseline dengue serostatus. Seropositivity was defined as MNT neutralizing titer ≥10 against at least one dengue serotype. Limit of detection (LOD): MNT=10 for NAb responses.

Supplementary Figure 4. Seropositivity rates (error bars: 95% confidence intervals) against individual DENV serotypes by baseline serostatus (per protocol set)

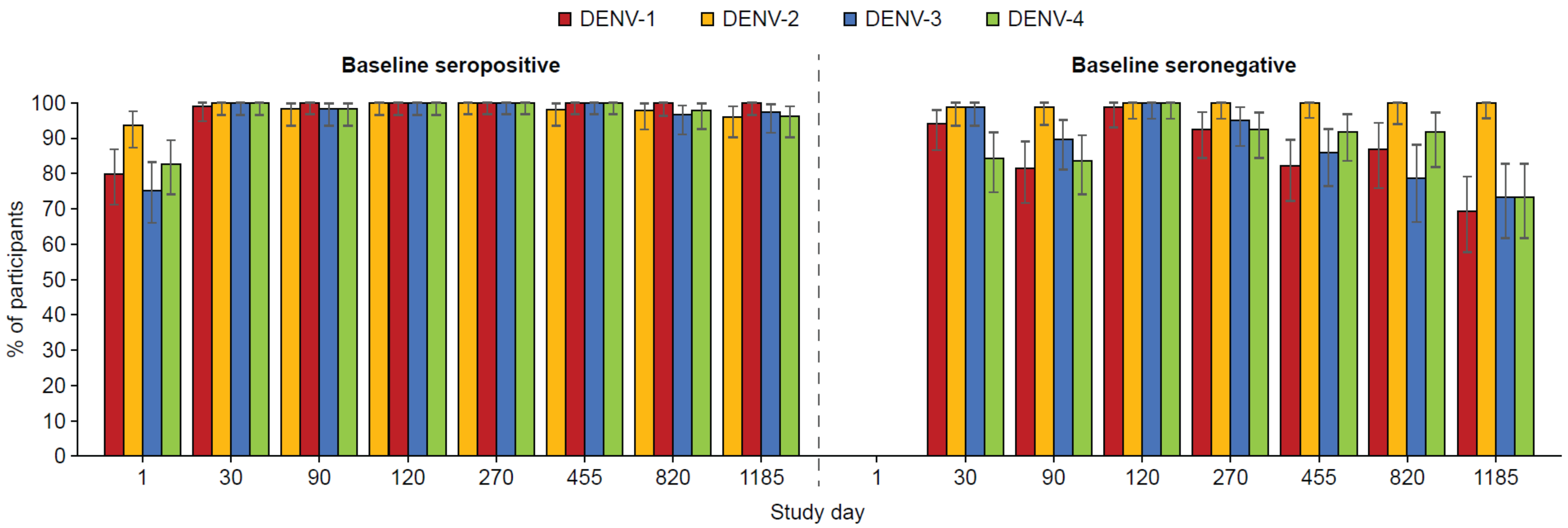

Day	1	30	90	120	270	455	820	1185
Baseline seropositive participants, n	109	102	109	104	107	108	93	102
Baseline seronegative participants, n	86	83	86	78	80	84	61	78

The terms “seropositive” and “seronegative” refer to baseline dengue serostatus. Seropositivity was defined as MNT neutralizing titer ≥10 against at least one dengue serotype

Supplementary Figure 5. Expansion of cellular and neutralizing antibody responses in baseline seropositive and seronegative vaccinees following virologically-confirmed dengue (VCD; per protocol set).

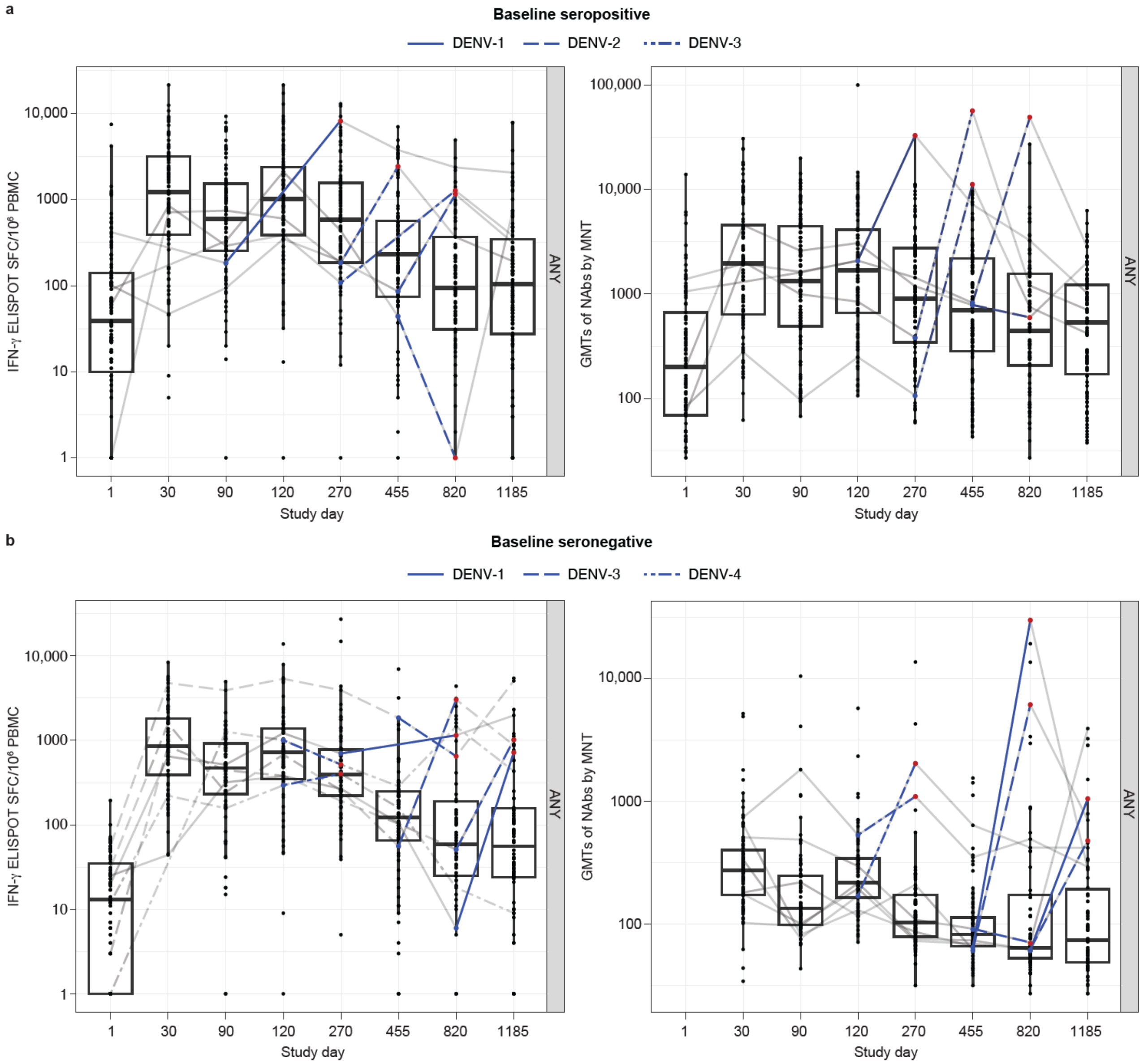

ELISPOT, enzyme-linked immunospot; GMT, geometric mean titer; IFN-γ, interferon-gamma; MNT, microneutralization test; NAb, neutralizing antibody; PBMC, peripheral blood mononuclear cells; SFC, spot forming cells.

Magnitude of T cell responses (number of SFC/10^6 PBMCs) for IFN-γ ELISPOT to any DENV peptide pool (DENV-2 NS1; DENV-1, -2, -3, and -4 NS3, and DENV-1, -2, -3, and -4 NS5) is shown on left and GMT of neutralizing antibodies to any DENV serotype are shown on right. Box and whisper plots represent the distribution of cellular/antibody responses for the entire study population at each time point. Blue lines show the changes in cellular/antibody response for individual participants with VCD, with a blue dot representing the visit prior to onset of

VCD and the red dot representing the visit after onset of VCD. Boxplot elements: center line, median; box limits, upper and lower quartiles; whiskers, 1.5x interquartile range; points, outliers.

Limit of detection for IFN-γ ELISPOT (LOD): 5 SFC/10^6 PBMC. The terms “seropositive” and “seronegative” refer to baseline dengue serostatus. Seropositivity was defined as MNT neutralizing titer ≥10 against at least one dengue serotype. Limit of detection (LOD): MNT=10 for NAb responses.

Supplementary Figure 6. Cellular and neutralizing antibody responses against any and individual DENV serotypes in baseline seropositive and seronegative participants with virologically-confirmed dengue (VCD; per protocol set).

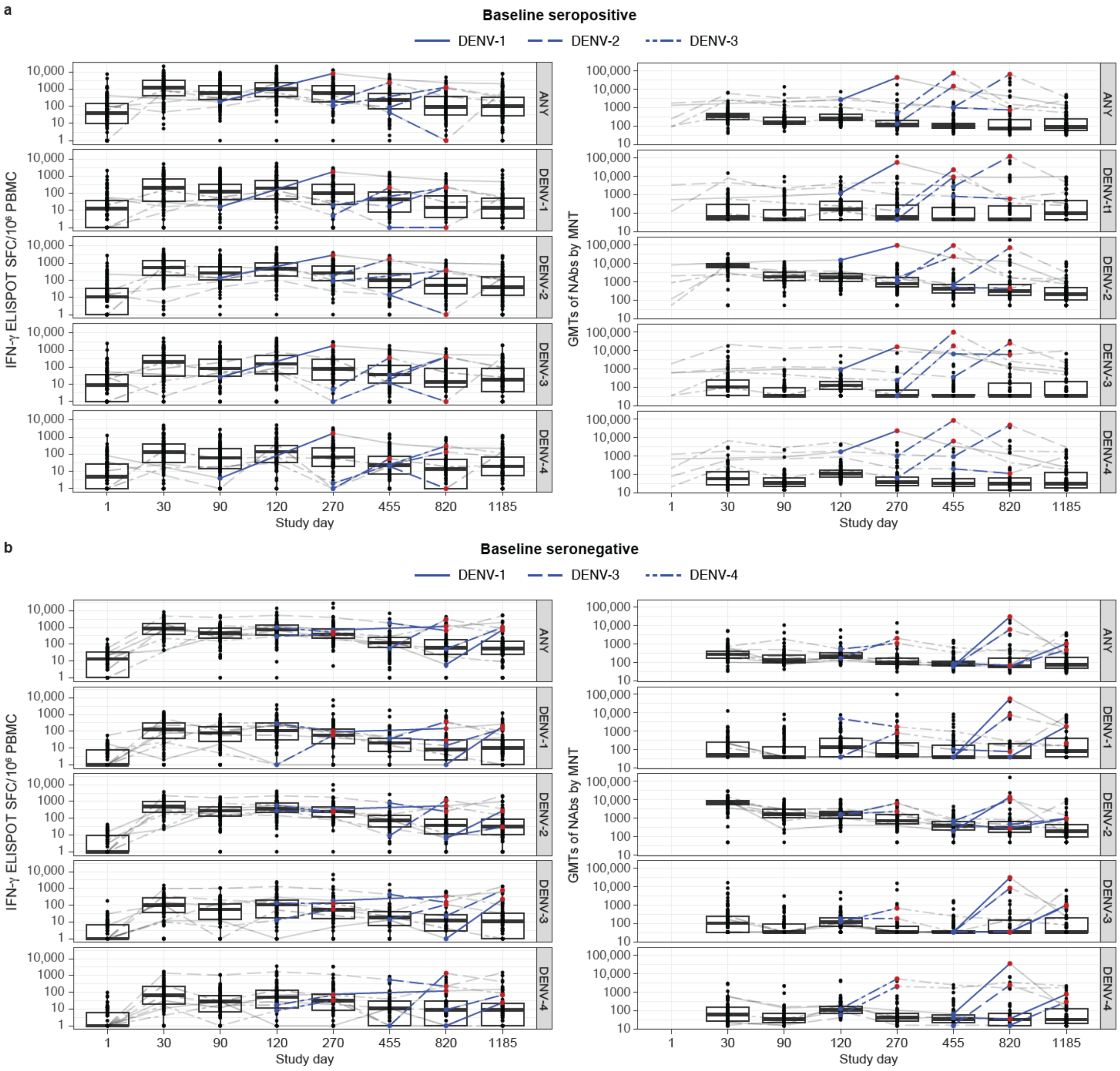

ELISPOT, enzyme-linked immunospot; GMT, geometric mean titer; IFN-γ, interferon-gamma; MNT, microneutralization test; NAb, neutralizing antibody; PBMC, peripheral blood mononuclear cells; SFC, spot forming cells.

T cell response magnitude (number of SFC/10^6 PBMCs) of IFN-γ ELISPOT to any DENV peptide pool (DENV-2 NS1; DENV-1, -2, -3, and -4 NS3, and DENV-1, -2, -3, and -4 NS5) is shown on first panel followed by IFN-γ ELISPOT to serotype matched DENV peptide pools (DENV-1, -2, -3, and -4 NS3, and DENV-1, -2, -3, and -4 NS5). GMT of neutralizing antibodies to any DENV serotype are shown on second panel followed by GMT of neutralizing antibodies for each DENV serotype. Box and whisper plots represent the distribution of cellular/antibody responses for the entire

study population across serotypes at each time point. Blue lines show the changes in cellular/antibody response for individual participants with VCD, with a blue dot representing the visit prior to onset of VCD and the red dot representing the visit after onset of VCD. Boxplot elements: center line, median; box limits, upper and lower quartiles; whiskers, 1.5x interquartile range; points, outliers.

Limit of detection (LOD) for IFN-γ ELISPOT: 5 SFC/10^6 PBMC. The terms “seropositive” and “seronegative” refer to baseline dengue serostatus. Seropositivity was defined as MNT neutralizing titer ≥10 against at least one dengue serotype. Limit of detection (LOD): MNT=10 for NAb responses.

Supplementary Figure 7. Study design

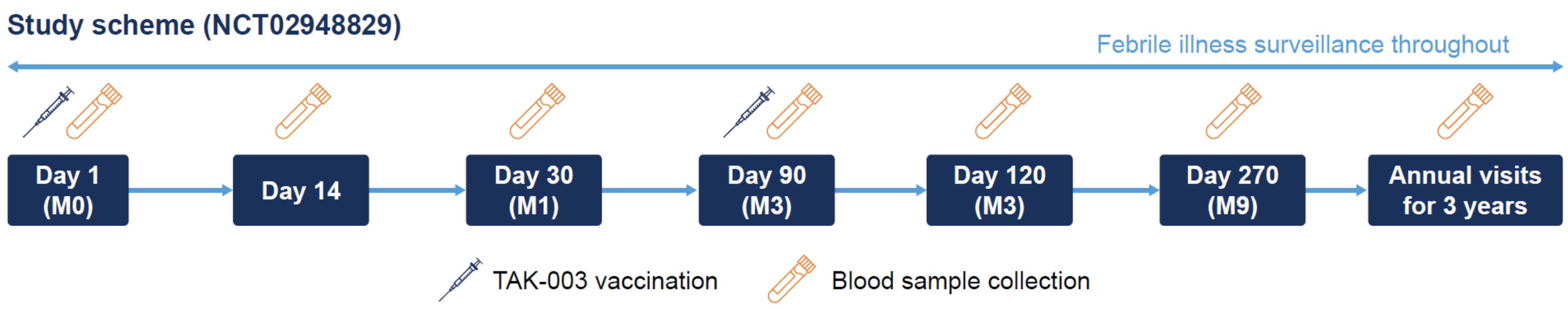

M, month

Supplementary Figure 8. Fluorescence-activated cell sorting (FACS) gating strategy for identification of CD4 and CD8 T cells, and cytokine-secreting cells

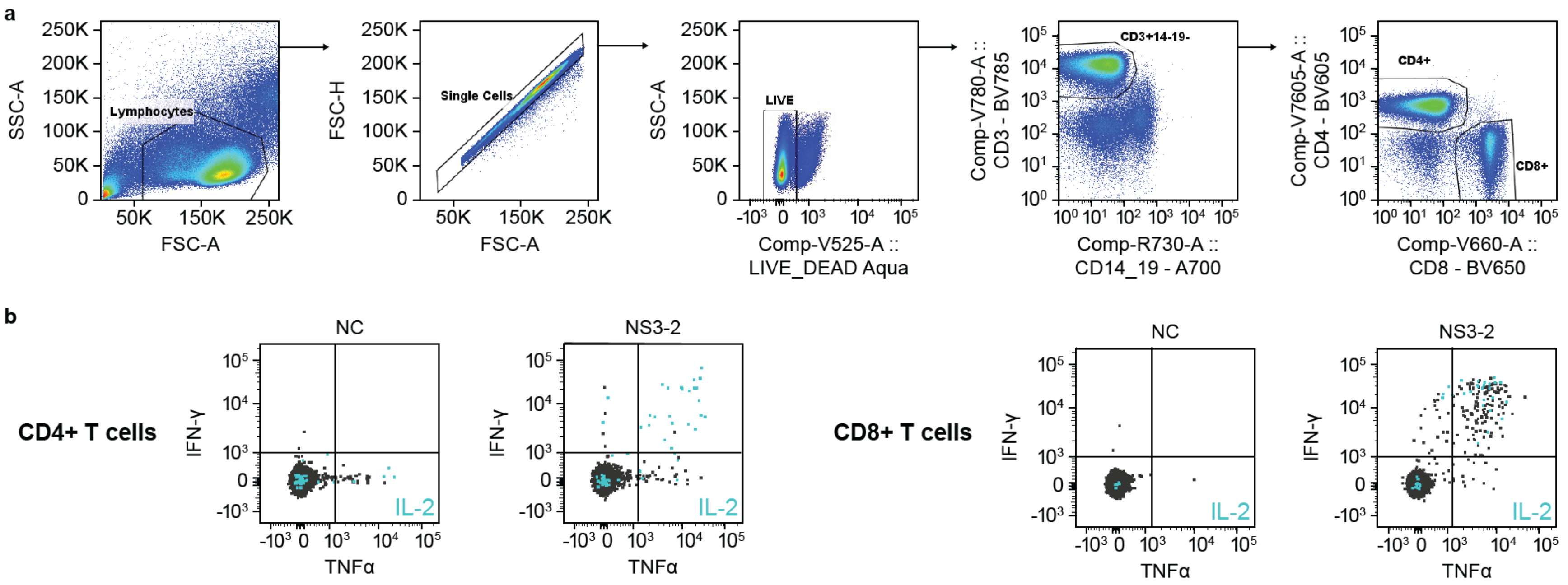

Supplement: Supplementary file 1 — Supplementary Materials [file 41541_2024_967_MOESM1_ESM.pdf]
